# Supplementary material for: Ectopic Expression of Grapevine Gene VaRGA1 in Arabidopsis Improves Resistance to Downy Mildew and Pseudomonas syringae pv. tomato DC3000 But Increases Susceptibility to Botrytis cinerea
Source: Int J Mol Sci. 2019 Dec 27;21(1):193. doi: 10.3390/ijms21010193 (PMC6982372; doi:10.3390/ijms21010193)
Supplement: Supplementary file 1 [file ijms-21-00193-s001.zip › ijms-626138 -revised-r2-supplementary/Supplementary Files/Table S1.docx]

**Supplementary Table 1.** Gene-specific primers used for qRT-PCR.

| Gene | Forward primer (5’-3’) | Reverse primer (5’-3’) |
| --- | --- | --- |
| *AtNPR1* | GCTCTGCTCGTCAATGGTTATC | GAGGAGTCGGTGTTATCGGTA |
| *AtEDS1* | TCATACGCAATCCAAATGTTTAC | AAAAACCTCTCTTGCTCGATCAC |
| *AtLox3* | TCTCCGTACAACAAGCGTTGG | GCGTCCGTCTAGCGCATTAAT |
| *AtPR3* | CGCTTGTCCTGCTAGAGGTT | GCTCGGTTCACAGTAGTCTGA |
| *AtActin1* | GTCTGGATTGGAGGGTC | TGAGAAATGGTCGGAAA |
| *VaRGA1* | AAGGAAAGTTGTGGGTGG | TTCGGATAAGTCTAAGGATA |
| *VaActin1* | GATTCTGGTGATGGTGTGAGT | GACAATTTCCCGTTCAGCAGT |
| *PVa* | CCGGTCCACCCTAAAGTAATC | CTAAAGCCGCGTAGAGATGG |
