# Supplementary figures and images for: Ectopic Expression of Grapevine Gene VaRGA1 in Arabidopsis Improves Resistance to Downy Mildew and Pseudomonas syringae pv. tomato DC3000 But Increases Susceptibility to Botrytis cinerea
Source: Int J Mol Sci. 2019 Dec 27;21(1):193. doi: 10.3390/ijms21010193 (PMC6982372; doi:10.3390/ijms21010193)

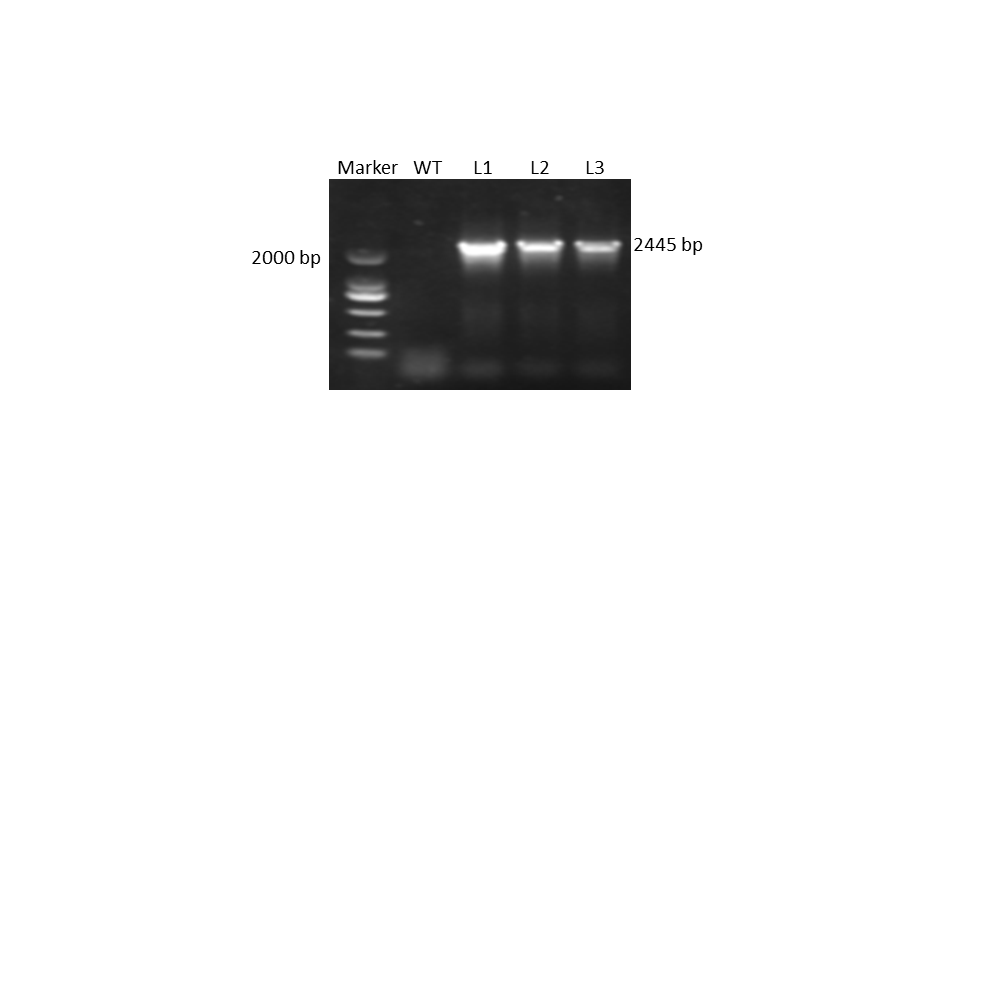

Supplement: Supplementary file 1 [file ijms-21-00193-s001.zip › ijms-626138 -revised-r2-supplementary/Supplementary Files/Supplementary Figure 1.tif]

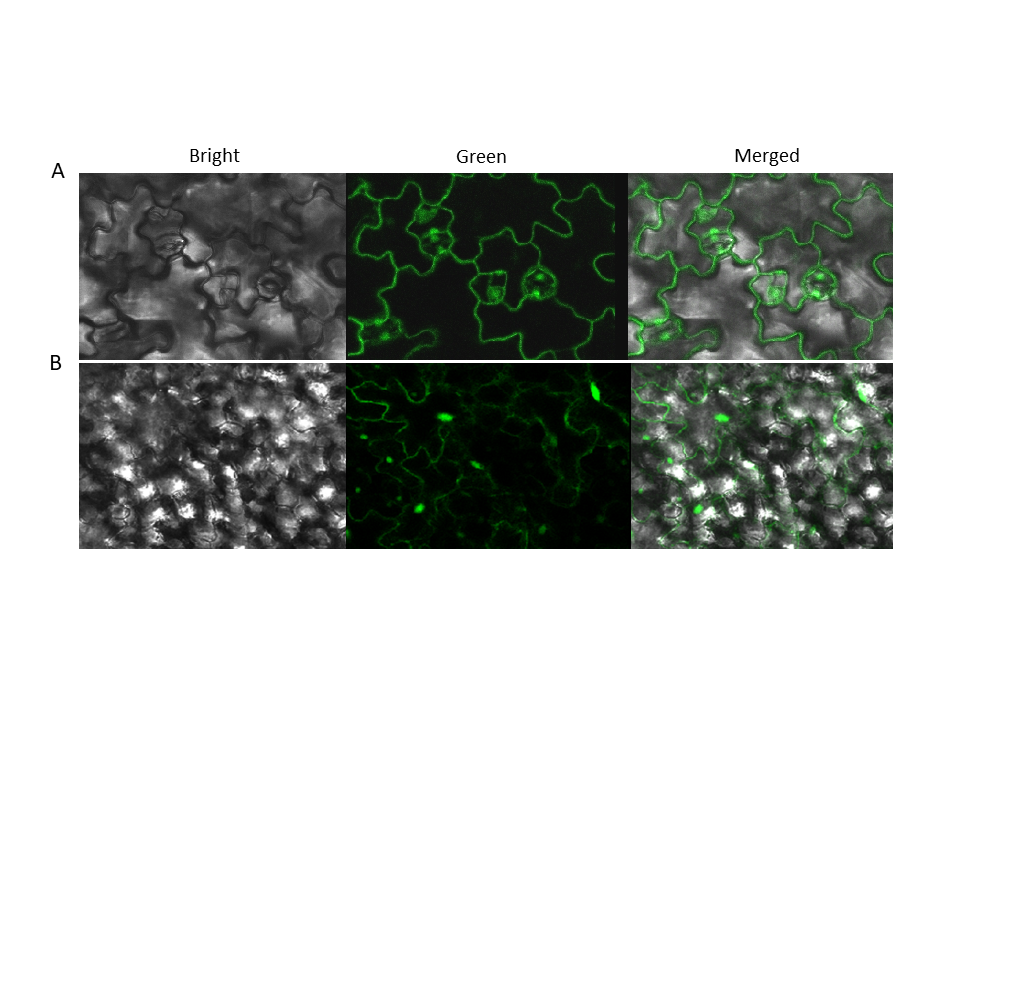

Supplement: Supplementary file 1 [file ijms-21-00193-s001.zip › ijms-626138 -revised-r2-supplementary/Supplementary Files/Supplementary Figure 2.tif]

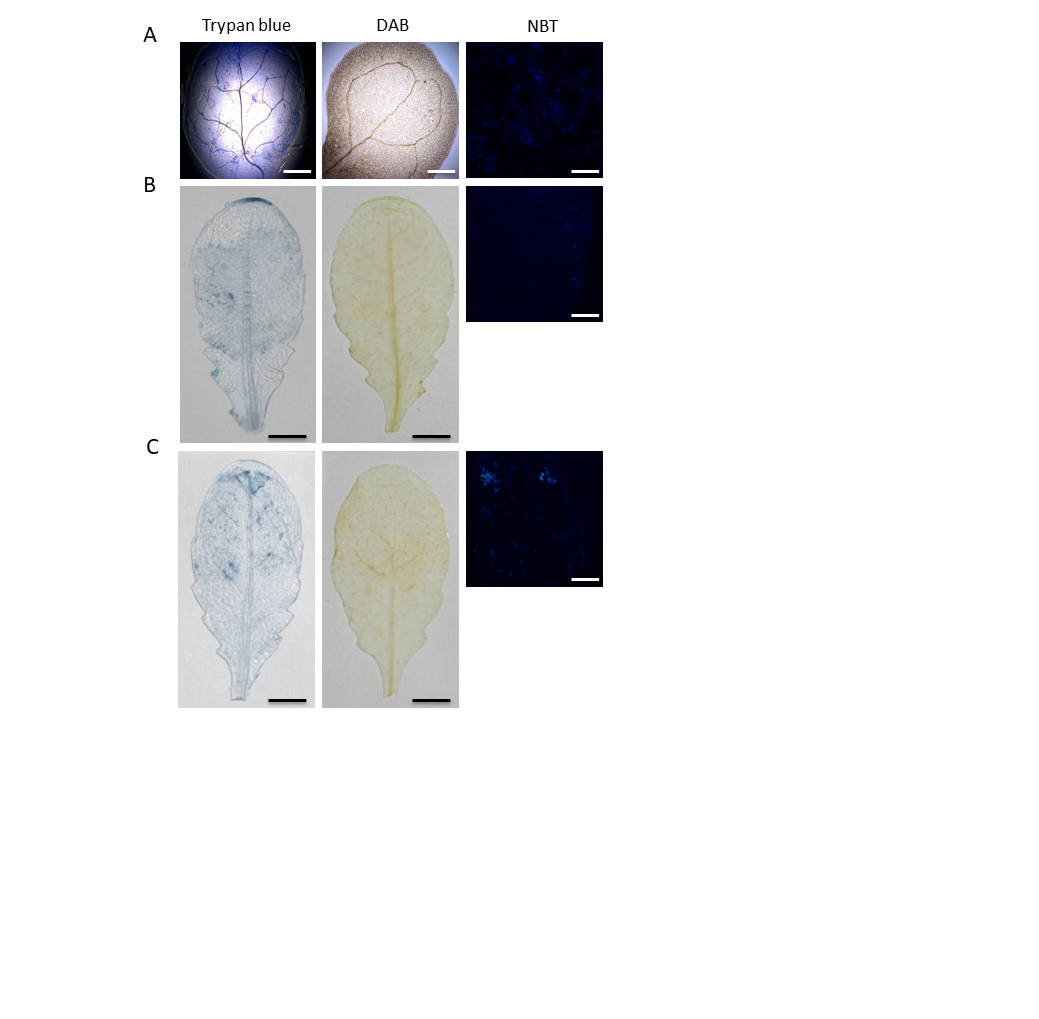

Supplement: Supplementary file 1 [file ijms-21-00193-s001.zip › ijms-626138 -revised-r2-supplementary/Supplementary Files/Supplementary Figure 3.tif]

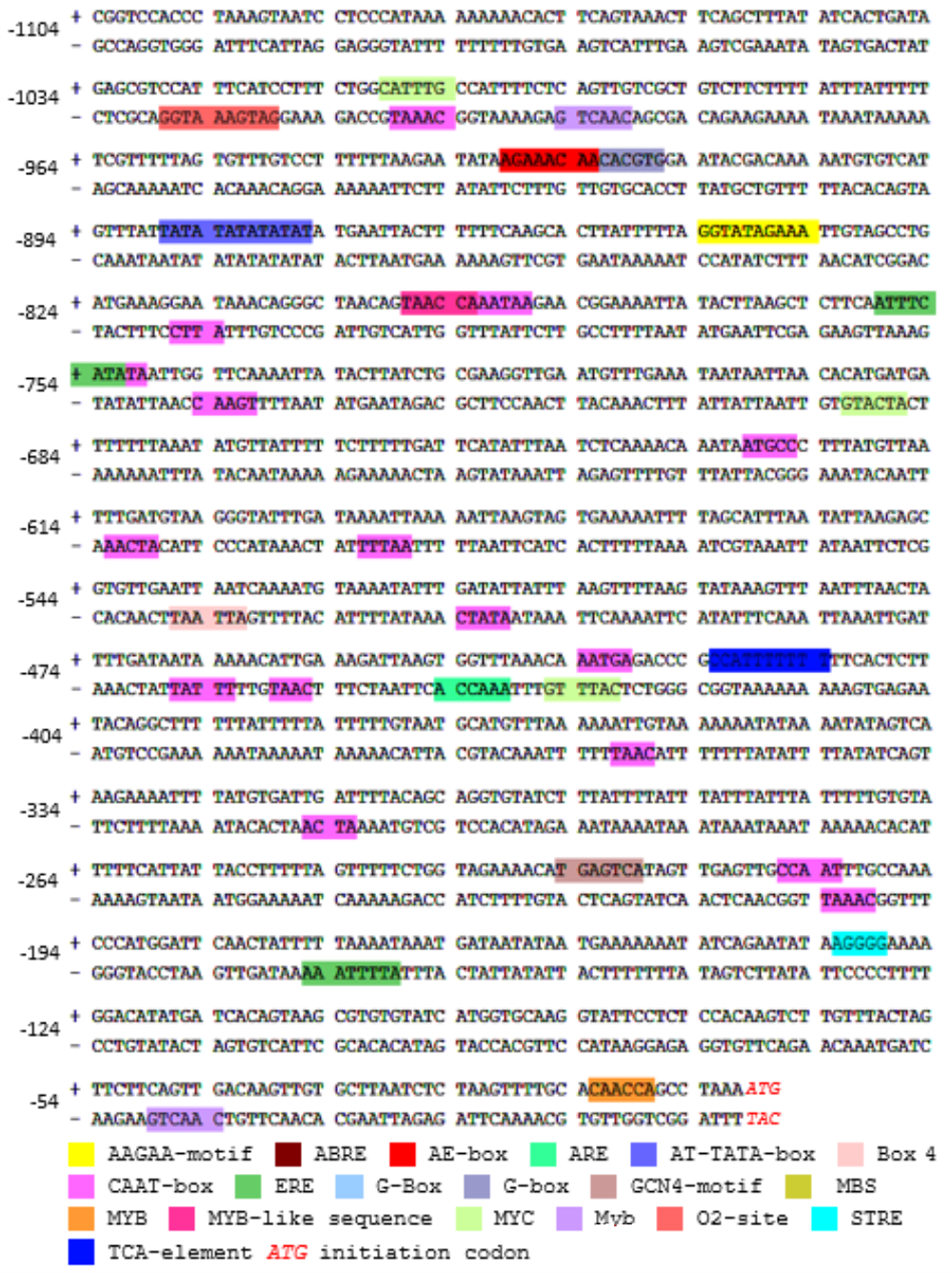

Supplement: Supplementary file 1 [file ijms-21-00193-s001.zip › ijms-626138 -revised-r2-supplementary/Supplementary Files/Supplementary Figure 4.tif]
